# Machine learning to analyse omic-data for COVID-19 diagnosis and prognosis

**DOI:** 10.1186/s12859-022-05127-6

**Published:** 2023-01-06

**Authors:** Xuehan Liu, Md Rakibul Hasan, Khandaker Asif Ahmed, Md Zakir Hossain

**Affiliations:** 1 Biological Data Science Institute, Australian National University, Canberra, ACT 2601 Australia; 2 BRAC University, Dhaka, 1212 Bangladesh; 3 Commonwealth Scientific and Industrial Research Organisation, Canberra, ACT 2601 Australia

**Keywords:** COVID-19 diagnosis, Severity, Multi-omics, Machine learning, Autoencoder

## Abstract

****Background**:**

With the global spread of COVID-19, the world has seen many patients, including many severe cases. The rapid development of machine learning (ML) has made significant disease diagnosis and prediction achievements. Current studies have confirmed that omics data at the host level can reflect the development process and prognosis of the disease. Since early diagnosis and effective treatment of severe COVID-19 patients remains challenging, this research aims to use omics data in different ML models for COVID-19 diagnosis and prognosis. We used several ML models on omics data of a large number of individuals to first predict whether patients are COVID-19 positive or negative, followed by the severity of the disease.

****Results**:**

On the COVID-19 diagnosis task, we got the best AUC of 0.99 with our multilayer perceptron model and the highest F1-score of 0.95 with our logistic regression (LR) model. For the severity prediction task, we achieved the highest accuracy of 0.76 with an LR model. Beyond classification and predictive modeling, our study founds ML models performed better on integrated multi-omics data, rather than single omics. By comparing top features from different omics dataset, we also found the robustness of our model, with a wider range of applicability in diverse dataset related to COVID-19. Additionally, we have found that omics-based models performed better than image or physiological feature-based models, proving the importance of the omics-based dataset for future model development.

****Conclusions**:**

This study diagnoses COVID-19 positive cases and predicts accurate severity levels. It lowers the dependence on clinical data and professional judgment, by leveraging the utilization of state-of-the-art models. our model showed wider applicability across different omics dataset, which is highly transferable in other respiratory or similar diseases. Hospital and public health care mechanisms can optimize the distribution of medical resources and improve the robustness of the medical system.

## Introduction

### Background

Healthcare centres urgently need novel technologies to identify symptoms for accurate diagnosis and severity prediction as a critical area. Coronaviruses, particularly those of the genus beta coronavirus (e.g. Middle East Respiratory Syndrome Coronavirus, aka MERS-CoV and Severe Acute Respiratory Syndrome Coronavirus, aka SARS-CoV), are highly pathogenic agents of respiratory disease whose highly variable genetic diversity and diverse host-adaptive features make them lethal and devastating worldwide [1]. This diversity has led to the global spread and transmission to millions of people, with more than 6.4 million deaths from COVID-19 (SARS-CoV-2) worldwide [2]. The clinical presentation of COVID-19 involves a wide range of symptoms and disease trajectories, and the clinical course of SARS-CoV-2 infection ranges from an asymptomatic state to a life-threatening infection with a high degree of variability and host adaptation [3]. Therefore, in addition to research into vaccines for the prevention of COVID-19 and specific drugs for treatment, it is imperative to develop tools for the diagnosis and prognosis of patient susceptibility for determining the status of patients, for rational clinical treatment, and for better management of health care resources.

Most current research on COVID-19 has focused on its epidemiological and clinical characteristics [4, 5]. There are two main methods for diagnosing COVID-19 today: bioassays and clinical characterisation. The mainstream biological diagnosis method requires virus nucleic acid detection, such as the RT-PCR nucleic acid detection method. It has fast diagnosis speed and high accuracy but needs professional experimental equipment and researchers. Another mainstream method is to rely on the early symptoms of COVID-19. Fever, cough, and dyspnea are considered potential indicators of suspected COVID-19. The future severity condition of covid-19 patients are also scored and predicted mainly based on these indicators. The judgment in this method is direct, and the data is easy to obtain. Still, it can be affected by the numerous surrounding factors, and the accuracy and reliability are often compromised

Several bio-computing solutions, including machine learning (ML), are currently being developed for the diagnosis and severity prediction of COVID-19. Its’ high precision, high automation, and interpretability can benefit the public health system to mitigate COVID-19 effects, having great potential to interpret high-dimensional and complex datasets. Recently, several studies has explored different ML models for COVID-19 diagnosis and prognosis [6–8]. Brinati et al. [9] proposed ML model to detect the infection of COVID-19 based on routine blood, with accuracies ranging from 82 to 86%. Some studies have explored robust ML models based on patients’ conventional clinical data, disease history, epidemiological factors and other physiological characteristics to diagnose covid-19 [10, 11]. A study investigated the value of Chest-tomography (CT) images for covid-19 severity assessment and clinical outcome prediction [12]. The significant results of these studies proved the feasibility and clinical rationality of using ML method to diagnose and prognosis covid-19 patients.

While, there are several models and datasets for COVID-19 detection, there is a handful of dataset and models for predicting the severity of COVID-19. Predicting severity is an important task to identify the progression of the disease and direct severe patients to immediate medical support—which will ultimately reduce the mortality of the patients. Several biological data, such as peripheral blood samples and blood oxygen index, have been used as essential indecs to predict the severity of COVID-19 patients. Recent evidence suggests that the severity of the disease depends largely on host factors. Many studies about the pattern of death with infection reveal surprising results: the cause of death cannot usually be attributed to the pathogen or the direct effects of any associated toxins it produces. Instead, it results from a systemic inflammatory response by the host’s immune system; in a way, our immune system destroys our health [13]. These studies support the need to understand individual responses at the molecular level better. With the deepening of research on COVID-19, medical research institutions have produced a large number of omics data [14]; however, the research on the influence of COVID-19 on the host’s omics data is not adequate.

Initial studies were based on single-omics data; for example, Macías-García et al. [15] proposed a limited set of related genes to characterise breast cancer recurrence based on DNA methylation data using autoencoder and random forest (RF) classifier. Park et al. [16] used large-scale gene expression and DNA methylation data to predict Alzheimer’s disease by a neural network model, showing that such integration of DNA methylation data improves prediction accuracy. Multi-omics provides a better representation of the host’s physiological condition and extent of disease, and several experiments have confirmed the feasibility of histology-based disease diagnosis and prognosis. A study analysed serum samples of critical and non-critical COVID-19 patients [17]. They trained ML models using proteomic and metabolomic measurements and validated the classification results in an independent cohort, revealing characteristic serum in critically ill COVID-19 patients’ protein and metabolite changes that can be used to select potential blood biomarkers for severity assessment. It demonstrates the great potential of multi-omics data for COVID-19 diagnosis and prognosis.

At present, the research on covid-19 using ML mainly focuses on the clinical information of covid-19 patients. Often the host-response, e.g. early stage of COVID-19, at the physiological level is undetectable, which can be captured easily at the molecular level. The host response at the molecular level is vital and often provides numerous features for ML models to train on. Besides, to make the scenerio even more complicated, the responses often modulated several bio-molecules and underlying pathways—which can not be answered by analyzing a single omics study. There is a scarcity of ML models for omics-based dataset to detect covid, and extend the modeling towards developing severity prognosis modelling.

The aim of our research is to apply the value of a large number of omics data to develop high-precision diagnostic and prediction tools for covid-19. We built a covid-19 diagnosis and severity models, based on single and multi-omic data. The study showed potential to be applied in diverse omics datasets, related to different respiratory diseases.

## Results and discussion

### COVID-19 diagnosis and prognosis using single-omics data

**Fig. 1:**
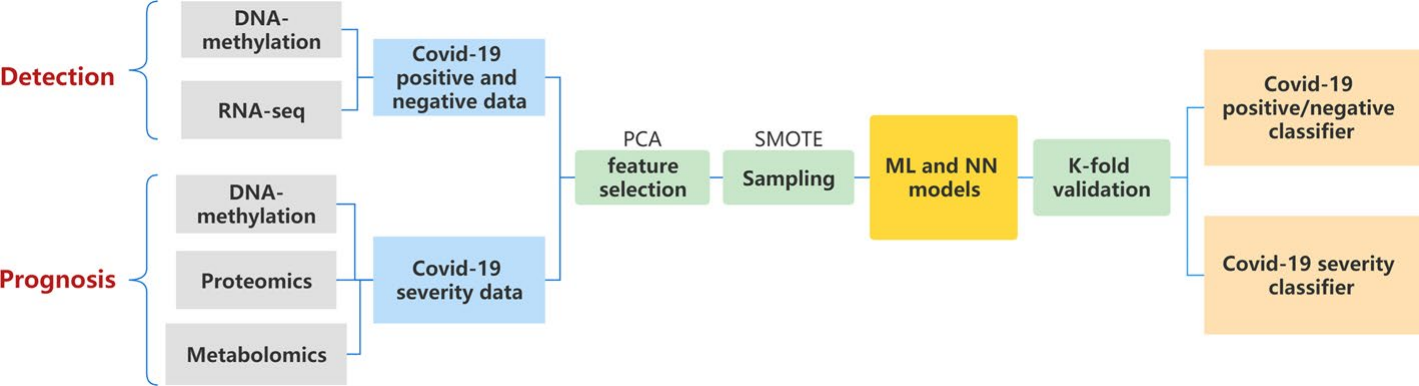
The workflow of diagnosis and prognosis of COVID-19 using single-omic data

First we explored the single omics dataset for diagnosis and severity prediction model. We collected DNA-methylation [3], RNA-seq or Transcriptome data [18], Metabolomics [19] and proteomics data [17] from different studies. Among these dataset, only DNA-Methylation datase has both anotations of presence/absence and severity levels. The RNA-seq dataset lacks distinct classification standards for the severity of COVID-19 patients, so we only performed COVID-19 negative and positive classification tasks on this dataset. The Proteomics and Metabolomics dataset lacks accurate identification of COVID-19 negative and positive patients, so we performed COVID-19 severity prediction and not the negative versus positive classification on this dataset. The workflow of this single omics experiment is shown in Fig. 1.

#### COVID-19 diagnosis: negative and positive classification

We used several single omic data to build ML models for COVID-19 negatives and positives classification. We first used DNA methylation data and RNA-seq data. We used 5-fold cross-validation on a total of 128 and 54 available samples in the DNA methylation and RNA-seq datasets, respectively, so the total number of samples tested in each fold was around 25.6 and 10.8 in these two datasets.

Table 1 presents AUC, accuracy and F1-score of multiple models for the evaluation of negative and positive classification of COVID-19 using DNA methylation data and RNA-seq data without and with PCA processing. Overall, with PCA, we get better outcomes in all models. In terms of ML model, Linear regression (LR) performed best on both dataset, obtaining the best accuracy, F1-score and AUC of 0.91, 0.95 and 0.79 respectively for RNAseq data. While comparing performances of DNA-methylation and RNAseq data, it seemed, the performances were almost similar in “without PCA”, but after applying dimensional reduction, the performance of the RNA-seq model enhanced substantially, indicating the dimensional reduction method works better for RNAseq-data. On the other side, the KNN model performed the least, even though PCA improved its overall performance a bit. It might be due to the architecture of the KNN model, which calculates the distance from the input points to the sample points and receive a larger sample size of data from the unbalanced data. While using RNA-seq data, MLP achieved the best performance, with an accuracy of 0.99. LR also has a good prediction performance, having an F1-score of 0.98.

**Table 1:** The performance of different ML models and single omics dataset for COVID-19 detection

Model	DNA methylation						RNA-seq					
	Without PCA			With PCA			Without PCA			With PCA		
	AUC	Accuracy	F1-score	AUC	Accuracy	F1-score	AUC	Accuracy	F1-score	AUC	Accuracy	F1-score
SVM	0.63	0.59	0.67	0.54	0.81	0.89	0.63	0.59	0.67	0.95	0.98	0.98
RF	0.72	0.87	0.93	0.74	0.88	0.93	0.72	0.87	0.93	0.85	0.94	0.97
**LR**	**0.79**	**0.90**	**0.93**	**0.82**	**0.91**	**0.95**	**0.79**	**0.91**	**0.95**	0.95	0.98	0.98
KNN	0.51	0.30	0.27	0.61	0.56	0.64	0.51	0.30	0.27	0.93	0.89	0.92
RUS	0.75	0.87	0.92	0.81	0.87	0.92	0.75	0.87	0.92	0.93	0.94	0.96
**MLP**	0.54	0.61	0.62	0.88	0.93	0.93	0.54	0.61	0.62	**0.99**	**0.98**	**0.98**

Bold indicates best performing model

Since the data has high dimensionality, we applied PCA and trained the model under the same conditions. With a dimensionality of 100 after dimensionality reduction, the performance of the model improved. In the case of MLP, the dimensionality reduction resulted in a significant performance improvement of around 0.37 in RNA-seq data, as too high dimensionality can make MLP harder to fit or overfit due to the complexity of the network structure.

In addition to the performance improvement, the feature transformation significantly reduces time and machine costs when training the model. In the same environment, the training time and RAM usage before PCA processing is about three to five times that of the processed data. Therefore, an appropriate feature transformation approach can significantly improve the performance of the model and reduce the learning cost.

#### COVID-19 severity prediction

First, we predicted COVID-19 severity by DNA methylation data, proteomics data, and metabonomics data. In the processing of high-dimensional data, we analyzed and confirmed that PCA has a better optimization effect on the training of ML models on high-dimensional data. Therefore, we used PCA to reduce the dimension of single omics data used for COVID-19 severity classification, and the dimension selection was based on the number of training set samples.

On different data sets, there are different criteria for distinguishing the severity of COVID-19, and it is divided into different numbers of severity levels. In DNA methylation data, we have three different severity levels, and in proteomics data, there are two severity levels. Therefore, the confusion matrix cannot be used to directly compare the classification results between different omics data. So, we evaluated the performance of classifiers on different omics data through different evaluation indicators (Table 2).

**Table 2:** The performance of different ML models and single omics dataset for COVID-19 severity classification

Model	DNA methylation			Proteomic			Metabolomic		
	AUC	Accuracy	F1-score	AUC	Accuracy	F1-score	AUC	Accuracy	F1-score
SVM	0.54	0.56	0.40	0.52	0.54	0.41	0.50	0.55	0.30
RF	0.68	0.72	0.59	0.68	0.72	0.69	0.67	0.72	0.59
**LR**	**0.70**	**0.76**	**0.64**	0.60	0.56	0.64	0.58	0.53	0.54
KNN	0.60	0.59	0.51	0.52	0.59	0.48	0.60	0.49	0.51
RUS	0.48	0.53	0.42	0.52	0.59	0.48	0.39	0.35	0.32
**MLP**	0.69	0.73	0.74	**0.66**	**0.67**	**0.74**	**0.68**	**0.70**	**0.72**

Bold indicates best performing model

Our model got 0.70 accuracy for COVID-19 severity classification on different data sets. In DNA methylation data, the LR model achieved 0.76 accuracy. We have achieved a preliminary prediction of the severity of COVID-19 on the single group data. There was no significant improvement in the performance with various optimisation methods and parameter adjustments. The probable reason for such a low score is the limited features within a single omics data, while the prediction of COVID-19 severity involves complex molecular interaction among diverse molecules. Besides exploring other feature extraction methods and hyper-parameter optimizations, there is an enormous potential of improvement by integrating multi-omics data. Therefore, it directs us to mine multi-omics data for a more complete prediction scheme.

### COVID-19 severity prediction using multi-omics data

**Fig. 2:**
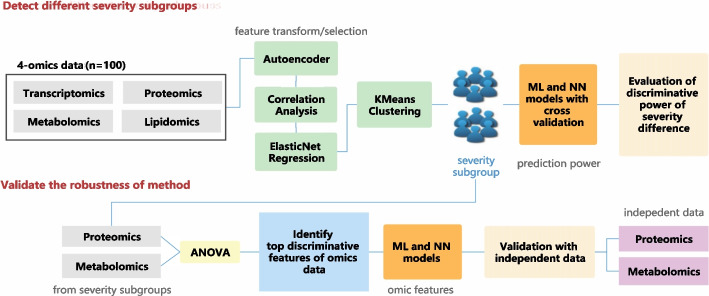
The workflow of COVID-19 severity prediction using multi-omic data

In this experiment, we used a multi-omics dataset, containing transcriptomics, protemics, metabolomics and lipidomics [20]. The dataset also contains annotations for both COVID-19 detection and severity levels based on hospital admission. The workflow of this experiment is shown in Fig. 2.

#### Severity subgroups

In order to classify and predict the severity of COVID-19 cases using multi-omics data, we used 100 samples obtained containing their 4-omics data. Each sample had transcriptomic, lipidomic, proteomic, and metabolomic data. For these 100 samples, we first preprocessed (discussed earlier) and obtained 13,263 features for transcriptomics, 647 features for lipidomics, 517 features for proteomics, and 105 features for metabolomics.

We used an autoencoder model, taking the stacked matrix of the 4-omics data as the input and output of the model, and then extracted the 500 nodes of the bottleneck layer as the result of the feature transformation. As the performance of the autoencoder is strongly influenced by the environment, the hardware environment, including RAM and GPU resources, we, conducted three iterations of the model to ensure the stability of the experimental results.

For each transformed feature, we performed a one-way correlation analysis. As the features generated by the autoencoder were not fixed, by analysing the Kendall correlation between the features and HFD-45 (hospital-free days at day 45), we retained an average of 455 features after three iterations and penalised them by elastic network regression to obtain a final sample of 42 features for clustering.

After clustering, we got two obvious subgroup by their distribution. We name the subgroups by G1 and G2. By looking at the clinical data of the two subgroups, we analysed the severity differences between the two subgroups obtained by clustering. We reduced the dimension of the sample into two dimensions by PCA. The difference in severity between the two subgroups was clear, and it was found that the severity of G1 was higher than that of G2 because the HFD-45 value of G1 was much lower than that of G2. To assess the validity of the clustering results, we compared each pair of sample combinations between the two subgroups. The predicted and actual results are consistent if the HFD-45 value of the sample from the subgroup with less severity is greater than the other comparison sample.

By comparing the values of HFD-45 between the two subgroups, we observed that the subgroup labels generated by clustering obtained a good agreement with the c-index of 0.76. At the same time, several indicators showed significant differences between these two subgroups, with a mean HFD-45 of 15.87 and 27.38 for first and second group. The considerable variability between the two means indicates a good differentiation in COVID-19 severity between the two subgroups. Therefore, the generated clustering labels effectively reflect the severity of COVID-19, with good discrimination between the different clinical stages of the case sample.

#### Severity prediction

A good severity classification cannot be achieved by using a single indicator as a classification label, so we cluster the samples and then performed supervised learning. We used the feature-processed sample data as training data, and the clustered 0–1 labels and HFD-45 median values as learning labels for the supervised models. We constructed SVM, RF, LR, KNN, RUS, and MLP models, obtained average results using 5-fold cross-validation, and repeated the experiments 3 times to ensure stability. The AUC, accuracy, F1 score, and c-index are shown in Table 3.

**Table 3:** The evaluation result of multiple machine learning models using clustering labels and HFD-45 median to predict COVID-19 severity

Model	Clustering labels				HFD-45 median		
	AUC	Accuracy	F1-score	c-index	AUC	Accuracy	F1-score
SVM	0.96	0.96	0.96	0.75	0.54	0.54	0.58
**RF**	**1.0**	**1.0**	**1.0**	**0.76**	0.73	0.73	0.74
**LR**	0.82	0.82	0.79	0.68	**0.85**	**0.84**	**0.84**
KNN	0.98	0.98	0.99	0.72	0.66	0.66	0.64
RUS	0.99	0.99	0.99	0.75	0.80	0.79	0.81
MLP	0.65	0.65	0.78	0.64	0.65	0.65	0.78

Bold indicates best performing model

We have observed that the labelled subgroup generated using the clustering training performed superior on several models. The RF and RUS models achieved significantly good performance here, as the labels were clustered by K-means, whose decision process is to select close category labels based on sample characteristics, similar to the decision process of ML models such as RF and KNN, and therefore more likely to achieve good performance. However, MLP was unable to fit the data better and therefore does not perform as well as the classical ML models.

While using cluster labels, we obtained a high prediction accuracy using the RF model. When training the model, we have achieved the correct classification for each data point through 5-fold cross-validation. The reason for high accuracy is that the sample size of test data after k-folding is small, and the model can predict all samples successfully. Secondly, the labels were obtained from individual clustering subgroups. Clustering labels affect the decision-making process of RF with high accuracy, which also indicates that, there is no overfitting problem within the prediction. However, we only obtained accurate predictions based on the subgroup labels, and a c-index of 0.76 reflects the prediction effect of the model on the sample ground truth. While using HFD-45 median values, we selected 100 samples with an overall median HFD-45 of 26 and a mean of 22. Since the presence of deceased cases, as well as undischarged cases with an HFD-45 value of 0, would have an impact on the overall distribution, we chose a median HFD-45 = 26 as the classification criterion and labelled patients as severe and less severe.

From Table 3, we observed that, for all models except LR, the classifier with the HFD-45 criterion can classify most of the samples correctly, but the performance drops nearly 20% compared to the classifier with subgroups as labels obtained by clustering, especially in the RF and the KNN models. If only the HFD-45 criterion is used on high-dimension samples, then the performance of the classification is lost as the key loci are not used as classification features while training. Secondly, as only the median of HFD-45 was used as the differentiation criterion, the difference between the omics data of patients close to the median and those far from the median was large. The way of classification ignored the variability of the samples within the interval. Therefore, the labels obtained using sample clustering achieved better performance on the classification task compared to a single clinical assessment criterion.

#### Performance of autoencoder with different input omics data

**Fig. 3:**
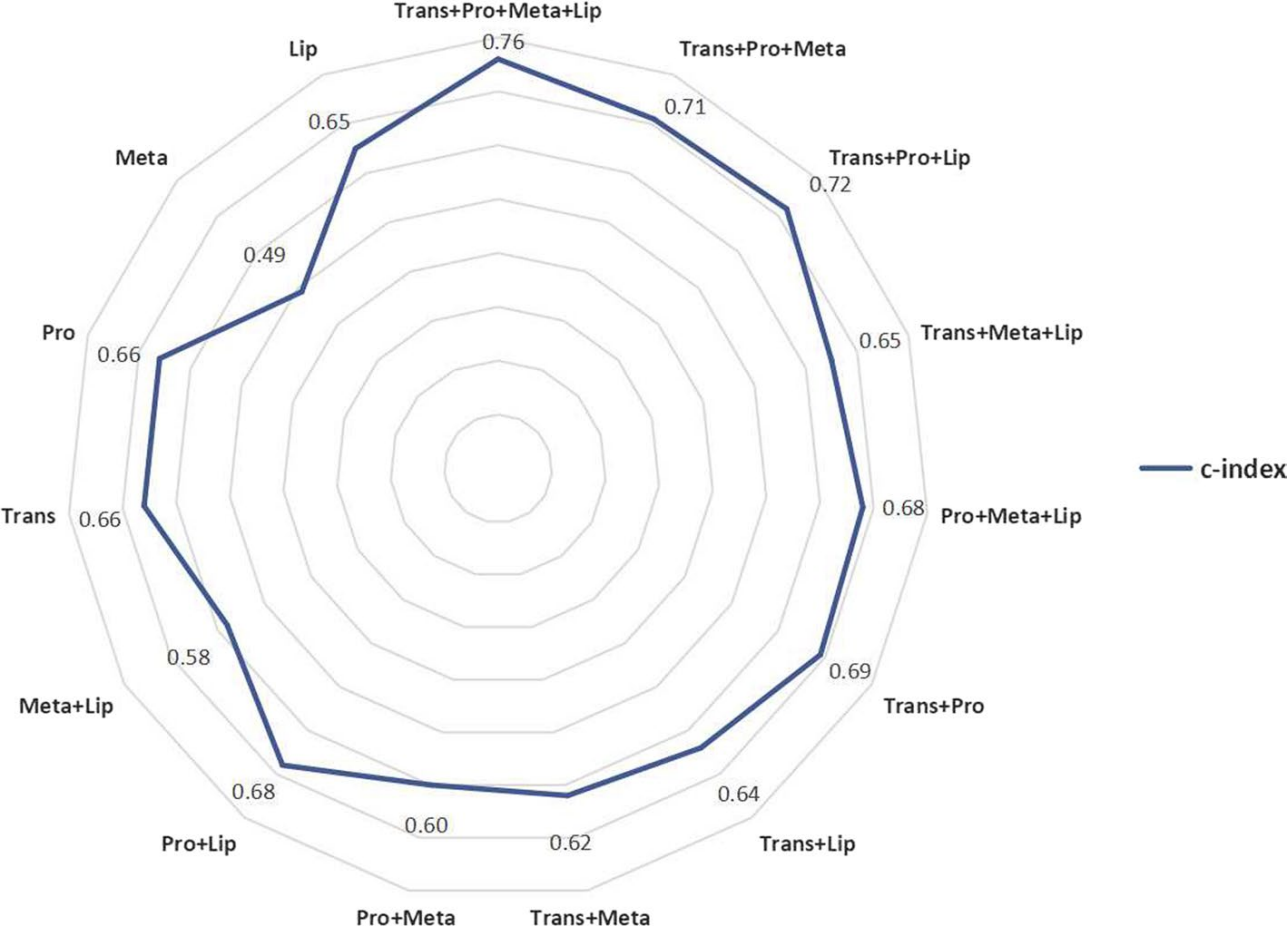
Rader graph of the c-index of different combinations of omics data in autoencoder. *Trans* transcriptomics, *Meta* metabolomics, *Pro* proteomics, *Lip* lipidomics

The use of different combinations of omics data as input features had a significant impact on the performance of the autoencoder. Figure 3 depicts a radar graph of the c-index of different combinations of omics data as input in our autoencoder model. It proves that the performance of the classification model based on multiple omics data is superior to that of single-omics data.

The autoencoder model using 4-omics achieves the highest c-index, reaching 0.72. This result indicates that the multi-omics data is more suitable for model construction than the single-omic data. The c-index of most combinations with transcriptomics is high, and a c-index higher than 0.6 indicates that the severity correlation between Transcriptomics and COVID-19 is stronger. The prediction performance with single Metabolomics showed the least value of 0.49. Within single omics dataset, the c-index of Proteomics and Transcriptomics dataset are similar (0.66). Interestingly, the c-index of the combination of Proteomics and Lipidomics reached 0.68, but the addition of Metabolomics reduced it to 0.62. The results showed that the prognostic ability of each omics data may not be additive.

#### Autoencoder versus its alternatives

To demonstrate the superiority of our Autoencoder-based deep learning model, we have conducted a comparative analysis of related models with our model. In addition to autoencoder-based multi-omics feature integration, traditional dimensionality reduction methods, such as principal component analysis (PCA) and cluster reduction methods, such as integral cluster analysis (iCluster) were also incorporated to evaluate the performance of multi-omics integration methods.

In the first method, we use PCA for feature transformation. Since our sample size was only 100 cases, we restricted PCA to reduce the feature dimension only to 100. After PCA converted the initial features to 100 principal components, Kendall correlation analysis was performed on the selected new features and only features with *p* values $<0.05$ were retained, resulting in the retention of 28 major components. The samples were clustered to obtain two COVID-19 severity subgroups and analysed with a c-index = 0.58. However, this method failed to significantly improve the consistency index between subgroups compared to the model using the autoencoder and showed poor performance in classification.

In the second method, we used iCluster for dimensionality reduction. Unlike PCA and autoencoder, iCluster analysis does not have to transform the initial omics features into new features, but rather groups the samples based on four omic data. Samples were clustered into groups directly based on the features from multi-omics data. iClusterPlus showed good predictive efficacy in both survival subgroups with a c-index = 0.62, but still, no significant improvement over the autoencoder-based model. The results of the comparison by c-index suggested that autoencoder-based multi-omics integration is superior to these alternative methods.

#### Verify the robustness of severity subgroups in independent data sets

**Fig. 4:**
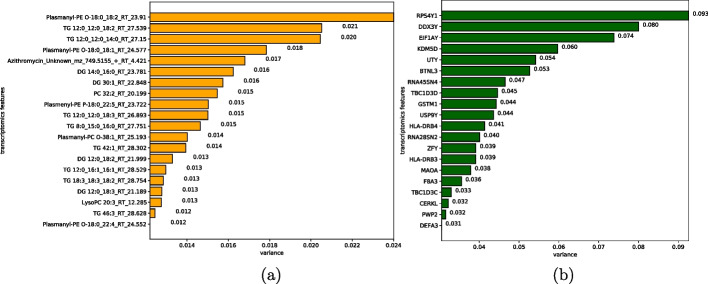
Top 20 difference features between subgroups. **a** Lipidomics. **b** Transcriptomics

**Fig. 5:**
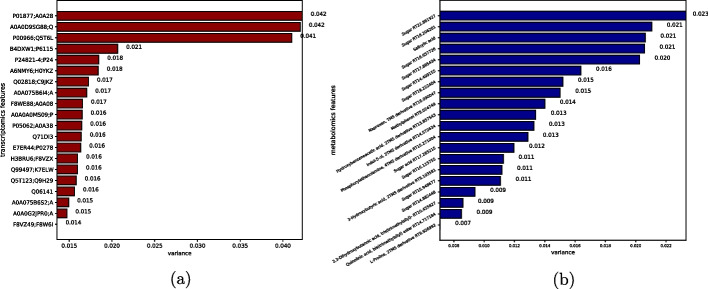
Top 20 difference features between subgroups. **a** Proteomics. **b** Metabolomics

To demonstrate the robustness of classification in predicting prognosis, we built two supervised classification models based on proteomics and metabolomics. We predicted the classification labels of samples from the validation dataset. We first performed Min-Max normalisation on each of the omic data. Then we selected the top N features of the most relevant to the clustering labels (obtained from the K-means) based on an analysis of variance (ANOVA). We set the default N value to 50 for proteomics and metabolomics, and 100 for transcriptomics and lipidomics. The top 20 significant features for each group are shown in figures: lipidomics and transcriptomics in Fig. 4, proteomics and metabolomics in Fig. 5.

The comparision of the top 50 important features within our model (proteomics and metabolomics) and another COVID-19 severity prediction study, Shen et al. [17] have shown several overlapping features. These results suggest that our multinomial data can be used to accurately predict the severity of any omics datasets related to COVID-19. Besides predictive modelling, these top features can be utilised in wider range of studies ranging from biomarker development to diagnose or prognose different levels of severity.

Besides, in the supervised learning models, independent omics data obtained results similar to training data. Proteomic data obtained the best performance on the RF model, with a c-index of 0.72. Metabonomics data obtained a c-index of 0.68 on the RUS model. The validation for robustness exemplifies the value of the information we obtained about the omics features for application on additional new dataset. The method we use not only yields good results on the training dataset, but also with the trained model, which can be used with the same type of omics data.

### Comparative analysis

In order to demonstrate the originality and validity of our work and its contribution to related work, we compare our results with those achieved by current related work in two ways: (1) omics data in any disease, and (2) omics and other data in COVID-19 disease only. We further discuss the advantages and shortcomings of each study, including our work.

#### Any disease prognosis/prediction with omics data

Past studies have used a combination of ML models and omics data to achieve an accurate prognosis for different diseases. We compared our achievement with several representative works in different evaluation methods, shown in Table 4.

**Table 4:** Comparative analysis with related work on disease prediction using omics data

References	Study target	Outcome
This study	COVID-19 severity prediction	Accuracy = 0.98
Sammut et al. [21]	Breast cancer therapy response prediction	Accuracy = 0.86
Macías et al. [15]	Breast cancer recurrence prediction	Accuracy = 0.68
Park et al. [16]	Alzheimer prediction	Accuracy = 0.82
This study	COVID-19 severity prediction	c-index = 0.75
Lee et al. [22]	Lung adenocarcinoma prognosis	c-index = 0.65
Zhang et al. [23]	Neuroblastoma prognosis	c-index = 0.71
Chaudhary et al. [24]	Liver cancer prognosis	c-index = 0.68

Compared with the current extensive schemes, the performance of our methodology is significantly higher. This result may be due to the severity of the influence on the omic components of patients with COVID-19. Generally, severe cancer patients will have extensive and complex physiological changes affected by the disease. It results in complex omics data, which is not easy to predict the prognosis of the disease. We compared our study with the prediction of Alzheimer’s disease by Park et al. [16] based on DNA methylation data and RNA-seq data. They put forward a multi-omics data scheme, by combining different sources, which significantly makes up for the lack of data in training prediction models. We also use the idea of multi-omics fusion, and experiments have proved that multi-omics data have better prediction performance than single omics data. In comparision with other studies, Sammut et al. [21] got 0.86% accuracy to predict the response to treatment of breast cancer, and discussed the effect of multi-omics fusion. Macías et al. [15] used autoencoder to predict re-occurence of breast cancer with single omic data (DNA methylation). Both of the studies used small dataset with high feature dimensions—which are often lack reliability. The work of Park et al. [16] combined and extracted features for omic data by biological information to predict Alzheimer’s disease. However, the used data are not from the same group of samples and the generated dataset can lead to the over-fitting problem.

Our current research are consistent with previous studies on liver adenoma [22] and neuroblastoma [23], which combine omics data with deep learning to improve the effect of feature processing of omics data. We have additionally feature-engineered using elastic network regression and optimised on a variety of ML models to obtain optimal performance. Compared with the subgroup classification and prediction of thymic carcinoma, liver cancer, and neuroblastoma, our study on COVID-19 obtained the highest consistency index.

#### COVID-19 prediction

At present, many studies have explored the diagnosis and prognosis of COVID-19 from multiple dimensions and directions. We have compared our research with some of these studies (Table 5).

**Table 5:** Comparative analysis with related work on COVID-19

References	Study target	Data used	Outcome
This study	Diagnosis	Omic	AUC = 0.95
			Accuracy = 0.988
Laguarta et al. [7]	Diagnosis	Cough signal	Accuracy = 0.985
Zakir et al. [1]	Diagnosis	Cough signal	Accuracy = 0.940
Khanday et al. [25]	Diagnosis	Clinical text	Accuracy = 0.962
This study	Severity prediction	Omic	Accuracy = 0.980
Overmyer et al. [20]	Severity prediction	Omic	Accuracy = 0.960
Shen et al. [17]	Severity prediction	Omic	Accuracy = 0.930
Cai et al. [12]	Severity prediction	CT image	AUC = 0.928
Aktar et al. [8]	Severity prediction	Blood samples	Accuracy = 0.930

We have observed that, compared with the ML prediction model based on CT images, cough signals and clinical texts, our model achieved better performance. It might be due to the wide features extracted from molecular dataset. Omics dataset are often able to detect the onset of diseases, which cannot be visible in physiological responses or behaviours. Previous studies [1, 7] used different physiological datasets, which is cost-effective, but often lack the reliability of onset of the disease. Khanday et al. [25] build models based on clinical text, which sophisticated feature engineering and can be improved further with deep learning methods. The biological significance of omics analysis can analyse the relationship between COVID-19 positive and host information, and carry out pathological research on COVID-19.

Overmyer et al. [20] proposed a web-based tool to predict covid-19 severity from diverse ethnicity, age and sex. Compared to their work, We combined the deep learning network in the feature extraction. We generate the severity label from clustering instead of the original label from their work. Even though we used same dataset, we got superior results. We optimised the feature processing of high-dimensional omics data and used a variety of ML models for training. Our research uses the c-index as the main index to measure the clustering performance. Compared with the classification model based on the severity classification of clinical indicator HFD-45, our prediction based on cluster tags has achieved higher accuracy. Therefore, we conclude that our model has improved the prediction performance of COVID-19. While, we couldn’t directly compare our results with Cai et al. [12] and Aktar et al. [8]—where they used CT-scan images and blood profiles respectively for training and testing of their models, our result has found omics-based models work considerably well than ct-scan or blood profiles, and training the model with omic data uses less computing source than dealing with image data. Moreover, among the omics-based models our model scored the top accuracy. So for future model development for disease prognosis and severity prediction, the omics-based datasets should need special consideration.

## Conclusion and future direction

We obtained better results for both the tasks of COVID-19 diagnosis and severity prediction. We implemented effective feature processing methods to solve the problem of high dimension and low sample size, which improves the performance of the model. To summarise the contribution of our work—we have first evaluated different single omics datasets with varying feature extraction methods and strategically progressed towards developing deep learning models for multi-omics dataset, related to COVID-19. Our study proved that, a combination of multi-omics dataset gave better predictive scores, compared to any score generated from any single omics data. We compared our models in terms of other diseases and also related to COVID-19 models, and found that our model outperformed all other disgnosis and severity prediction models. We give an effective COVID-19 diagnosis and prognosis method based on ML, which can be used in the healthcare system for infectious disease control and graded treatment, to some extent improving public health governance.

This research has generated several potential and promising directions for future work. First, we will investigate the ability to generalise the findings. Our current work can only guarantee validity in the four-omics features for limited number of samples. To contribute more to public health, we will further investigate other large-volume dataset, as well as increase the diversity of our dataset by selecting samples from different backgrounds. It will also be possible to enrich the type of omics data we use and obtain more extensive conclusions. We will use other ML models with complex structures for learning. Further, our current work is limited to the use of omics data for diagnosis, with a lack of experimentation with other medical data. In future work, we will add features like clinical information and patient health status to the input of the model. We analysed the Pearson correlation of clinical information and cluster label, where we observed that the label showed a strong correlation with multiple clinical information, such as Lymphocytes per cent. We would explore the optimisation and impact of additional data on current protocols. To investigate the use of the kind of combination that would allow for better diagnosis and prognosis of COVID-19 patients. Future research shall also focus on using pre-regulated and down-regulated expression genes from the omic signature for ingenuity pathway analysis. This approach would help investigate the value of our results for applications in biological and disease research.

## Method and experimental setup

### COVID-19 diagnosis and prognosis using single-omic data

This research first diagnosed COVID-19 (negative and positive classification), followed by severity prediction. We collected and processed multiple single omics data: DNA methylation, RNA-seq, Proteomics, and Metabolomics. Details on these datasets are included in Additional file 1 (omics data). We utilised ML models for diagnosis and prognosis. We tuned the model parameters and optimised the performance. Further, we optimised the dataset’s quality by sampling the data when the sample distribution was imbalanced. Next, we adopted a k-fold cross-validation approach to obtain average evaluation results.

#### Data preprocessing

Removing abnormal and erroneous data was the first step in data preprocessing. Five samples were removed from the proteomics and metabolomics dataset as they did not have proteomic data. For the treatment of null values in the dataset, we removed the feature with null values greater than 20% and performed zero value padding. As the data dimension was much larger than the number of samples, we reduced the dimension by principle component analysis (PCA) to efficiently train the models. For the samples, we reduced the dimension of features to the number of samples. We report the models’ performance, both with and without PCA, to demonstrate the role of PCA in the dataset. In our datasets, COVID-19 negative samples only account for a small part, resulting in severe imbalance data. To tackle data-imbalance problem random undersampling boost (RUS) was optimised using AdaBoost method. To compensate the imbalance and ML performance, we further applied SMOTE (synthetic minority oversampling technique) sampling method for data balance [26, 27].

#### Classification model

We implemented several ML models, namely support vector machine (SVM), random forest (RF), logistic regression (LR), K-nearest neighbourhood (KNN), random undersampling boost (RUS), and multilayer perceptron (MLP) algorithms. We adjusted the parameters of the MLP, including the number of connected layers and the number of nodes, to get better results for different dataset. The number of layers, nodes, dropout, and activation functions were decided using experiments, shown in (Table 6).

**Table 6:** The structure and parameters of a fully-connected MLP model on different data sets

Parameters	DNA methylation	RNA-seq	Proteomics	Metabolomics
Number of layers	3	2	2	2
Nodes of layers	[100, 64, 32]	[32, 28]	[24, 12]	[24, 12]
Dropout	0.3	0.0	0.0	0.0
Activation function	Relu	Relu	Tanh	Tanh

### COVID-19 severity prediction by multi-omic data

In this experiment, we used a unified dataset containing four omics data [20]. The dataset was collected from 128 adult SARS-CoV-2 virus-associated patients from Albany Medical Center in Albany, New York, USA. Blood samples of these patients were collected, and then transcripts, proteomics, metabolites, and lipids were measured from plasma. Description of the dataset can be found in Additional file 1. We performed feature selection with deep learning and statistical methods to select loci under critical conditions. Patients were classified into severity subgroups by unsupervised clustering. We determined their agreement with actual data and accuracy in supervised learning, validating their robustness with additional separate omics data.

#### Data preprocessing

We first removed the features with unknown labels in the metabolomics (45 unknown features) and lipidomics (2710 unknown features) analyses for data preprocessing. Then we cleaned the data by eliminating features and samples with NA values greater than 20% or zero values. The samples were then filtered to retain only patients with all four valid omic data, with some samples missing some omic data. Eventually, the final omic data was obtained for 100 patients.

The training dataset does not contain survival status and patient information over a continuous period, so we cannot directly rely on survival time as a severity measure. It requires us to select a value that reflects multiple indicators as an evaluation criterion. In this case, we used hospital-free days at day 45 (HFD-45) [20]. This score assigns a zero value (0-free days) to COVID-19 patients with a length of stay longer than 45 days or who died on admission. It gives a higher HFD-45 value to patients with a shorter length of stay and less severe disease. In terms of mean values, members of the healthy group had significantly higher HFD-45 values (mean = 32.3) than members of the COVID-19 group (mean = 22.0), with a *p* value of 0.004.

*Autoencoder* We used multi-omics datasets to build COVID-19 diagnosis and severity prediction models, which have high feature dimensions. We used autoencoder, correlation analysis, and elastic net regression to reduce the feature dimension and avoid over-fitting risk. In our work, the data of 100 patients were used as the input of the autoencoder using the preprocessed four-omics data. The four omics data were stacked into a matrix through sample scaling.

To build the autoencoder model, we chose *relu* activation function in input and hidden layers, and *tanh* activation function in the output layer. We used *binary crossentropy* loss function, which calculates the error between the input data and the output. L1 and L2 regularisation was used to reduce overfitting, where the L1 and L2 parameters were 0.001 and 0.0001, respectively. We used Python (v3.8.16), Sklearn (v1.0.2) and Keras (v2.9.0) to build an autoencoder and saved the value of the bottleneck layer node as a new feature extracted from the original four-omics data. We experimented with different combinations of hyperparameters while constructing the autoencoder model (Table 7).

**Table 7:** Experimented autoencoder structures with different combinations of parameters

Parameter	Model 1	Model 2	Model 3	Model 4
Number of layers	5	5	3	7
Connect layer size	2000	1000	1000	3000
Bottleneck size	500	500	300	500
Learning rate	0.001	0.001	0.0001	0.0001
Epoch	10	10	20	20
Dropout	0.2	0.5	0.2	0.5
c-index	0.76	0.68	0.62	0.59

We observed that performance decreases significantly if the number of hidden layers exceeds three, if the number of hidden layer nodes increases, or if the training epoch is large. However, with only one hidden layer, or too few hidden layer nodes, the model is less efficient at feature learning, affecting performance. Conversely, as the number of nodes in the bottleneck layer of an autoencoder model increases, the model’s performance can usually be improved, as the increased number of nodes in the bottleneck layer retains more feature information, which also helps the model to reduce the loss during training. Accordingly, we selected the autoencoder model having five layers. The first was the input layer, with three hidden layers in the middle, with the number of nodes of 1000, 500, and 1000, respectively. The middle layer was the bottleneck layer.

*Correlation analysis* After the autoencoder reduces the number of original features to 500 transformed new features obtained from the bottleneck layer, we choose an appropriate feature selection method to reduce the feature dimension further. We chose the Kendall rank correlation coefficient for cross-group correlation analysis to study the amount of linear correlation between variables.

We calculated the correlation and chose HFD-45 as the element selection standard to analyse the correlation of 500 features generated by the autoencoder. The elements with a *p* value $< 0.05$ were selected as the top-level characteristics significantly related to the severity of COVID-19.

#### Elastic net regression

We further selected the retained features from the correlation analysis in our work. We used elastic network regression. We used the values of the characteristics of all COVID-19 patients with *p* values $< 0.05$ in the correlation analysis as covariates and HFD-45 as the response variable. After several sets of experiments, we chose the parameter $\lambda$ of regularisation intensity of elastic network regression to be 0.5, the parameter $\alpha$ of balancing the intensity of L1 and L2 penalty terms to be 0.5, the threshold of convergence to be 0.001, and the maximum number of recursions to be 10000 times. We used this set of parameters to fit the complete data set.

#### Severity subgroup analysis

**Fig. 6:**
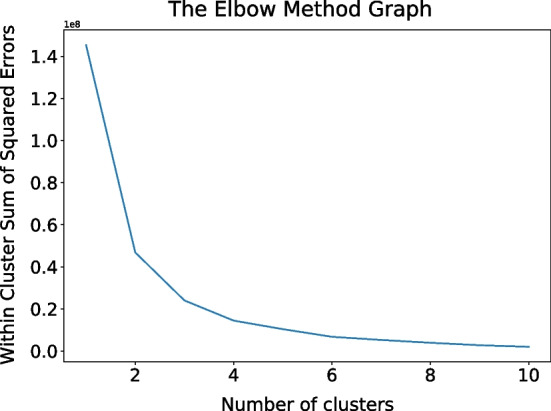
Elbow method folding diagram to determine the number of clusters K

In order to identify the best number of severity subgroups and obtain the key loci between groups affecting the severity, we used the K-means clustering algorithm for clustering. We determined the optimal number of clusters (K) using the elbow method [28], which uses the square of the distance between the sampling point and the cluster centroid in each cluster to give a series of K values. The sum of squared error (SSE) is used as a performance index. By drawing the K-SSE curve and finding the inflection point downward, the K value can be better determined. We built a K-means clustering model using *sklearn*. We observed SSE versus K graph for K = 1 to 20 (Fig. 6). It was found that when K = 2, the SSE value decreases significantly. We, therefore, divided the sample into two COVID-19 severity subgroups.

#### Prediction model construction for severity

In order to clarify the robustness of survival subgroups, we used a series of ML models belonging to supervised learning algorithms to classify the severity of COVID-19. The classification labels used for these ML classifiers were determined by K-means clustering rather than using a single clinical feature. In order to check the effectiveness of the performance of labels and features generated by the autoencoder, we performed 10-fold cross-validation to obtain the average evaluation results.

#### Verification of robustness of severity subgroups

To test the robustness of the subgroups obtained by clustering in terms of prognosis, we used independent proteomic and metabolomic datasets for validation. We obtained top-level different characteristics between the subgroups using analysis of variance (ANOVA). First, we performed Min–Max normalisation of the training set samples to avoid the effect of different feature data sizes on the ANOVA. We selected the top N features that are most relevant to the clustering labels (obtained from the K-means). Depending on the size and complexity of the dataset, we set the default N value to 50 for Proteomics and Metabolomics, and to 100 for Transcriptomics and Lipidomics.

We used ANOVA to collect the features with the greatest difference between the two severity subgroups in the training dataset. Among the features of proteomics and metabolomics, we selected the same features of the validation set (e.g., Q71DI3 in proteomics). We retained these features in the training dataset and generated a dataset for supervised training. To avoid differences in data platforms that result in data on the training dataset and the independent validation dataset, we used the StandardScaler method, which normalises the training dataset and the independent dataset based on the mean and standard deviation of the training dataset [29].

### Performance evaluation

Several evaluation metrics were used to evaluate individual model performances. We used Accuracy, F1-score, C-index and AUC as evaluation matrix. We used 10-fold cross-validation and the average test evaluation results are reported. It provides generalised results, which enhances the reliability.

In our work, the c-index is used as an important parameter to measure the classification accuracy of COVID-19 severity subgroups, and we redefined the calculation of the c-index based on the definition and calculation of the c-index. Concordance index (c-index), based on Harrell C statistics [30], is similar to AUC. It is calculated by randomly forming pairs of all the study subjects in the data under study. The c-index usually occurs between 0.5 and 1 (the probability of agreement and disagreement is exactly 0.5 for any pairwise random case). A value of 0.5 is complete disagreement, which means that the model has no predictive effect, and 1 is complete agreement, which means that the model’s predicted outcome is precisely the same as the actual one. Besides, we used clustering labels and HFD-45 median to predict the severity in four-omics dataset.

### Hardware setup

The experiments are conducted on a CPU with 25 GB RAM and an NVIDIA K80 GPU with 10 GB of memory on a Windows operation system. The work was implemented in Google Colab, with Python (v3.8.16), and other supporting ML libraries, including Tensorflow (v2.9.2), Sklearn (v1.0.2), and Keras (v2.9.0).

## Supplementary Information

**Additional file 1**: Omics dataset.

## Data Availability

All raw data, training sets, testing sets, and code used during the experimental analysis are available at https://github.com/lxhwww/machine-learning-for-covid-19. Publicly available datasets are used. The DNA-methylation and RNA-sq data are available on Gene Expression Omnibus with GSE174818 accession (https://www.ncbi.nlm.nih.gov/geo/query/acc.cgi?acc=GSE174818) and GSE171110 accession (https://www.ncbi.nlm.nih.gov/geo/query/acc.cgi?acc=GSE171110), respectively. The proteomics and metabolomics data are available at ProteomeXchange Consortium (https://www.iprox.org/), having project ID: IPX0002106000 and IPX0002171000 and used in [17]. The four-omic mass spectrometry raw files and the SQLite database file are available in the MassIVE database (https://doi.org/10.25345/C5F74G), having accession number: MSV000085703 and used in [20].
