# Supplementary material for: Machine learning to analyse omic-data for COVID-19 diagnosis and prognosis
Source: BMC Bioinformatics. 2023 Jan 6;24:7. doi: 10.1186/s12859-022-05127-6 (PMC9817417; doi:10.1186/s12859-022-05127-6)
Supplement: Supplementary file 1 — Additional file 1: Omics dataset. [file 12859_2022_5127_MOESM1_ESM.pdf]

Additional file 1: Omics dataset

1. Single-omics dataset

1.1. DNA methylation

Some of the available DNA methylation datasets of COVID-19 patients are too small or lack appropriate processing methods. We used a reliable and publicly available dataset (NCBI: GSE174818) [1]. , and collected by Joseph balnis's team from Albany Medical Center in Albany, New York. It contains 865859 DNA methylation points of 128 cases (102 COVID-positive and 26 COVID-negative) for moderate to severe respiratory failure presumably related to infection with SARS-CoV-2, collected from April 6 to May 1, 2020.

The dataset used a specific COVID-GRAM risk score for risk assessment to test whether the severity is related to DNA-methylation patterns in blood or not. The COVID-GRAM risk is synthesised from a series of clinical data and can reflect the patient's overall situation and disease progression. This score provides an estimate of the risk of critical illness for COVID-19 inpatients and helps to identify COVID-19 patients who may subsequently develop a critical illness. When this index is larger than a threshold, the patient's disease risk is higher, which is defined as a high-risk case. Gram risk score only evaluates patients with COVID-19 and does not apply to patients without COVID-19. The severity of COVID-19 patients was divided into three types: High GRAM (n = 55), Medium GRAM (n = 45) and Low GRAM (n = 2) [2].

1.2. RNA-seq

We collected RNA-seq dataset from a study of whole blood analysis, which is a body fluid composed of blood and plasma in severe COVID-19 patients and healthy blood donors (NCBI: GSE171110) and had access to clinical information records of cases [3]. This dataset was used to analyse whole blood gene expression. Samples were collected from 54 individuals in total, of which 10 were healthy, and 44 were severely COVID-19-affected.

1.3. Proteomic and metabolomic

We obtained proteomics and metabolomics of blood serum from a group of COVID-19 patients from an open-source study [4, 5]. The data was collected from 65 patients who visited Taizhou Hospital between January and March 2020. The serum samples were collected and were verified as COVID-19 positive or negative cases using PCR tests. For the positive sample, they set four stages of clinical characteristics: mild, typical, severe, and critical. We obtained a complete set of proteomic and metabolomic samples from the total samples. It contained protein matrix (791 features) and metabolite matrix (847 features) data from 31 COVID-19 patients (18 non-severe and 13 severe patients), with detailed patient descriptions.

2. Multi-omics dataset

For the subgroup division and prediction of COVID-19 severity, we used three datasets, namely 4-omics data containing transcripts, proteomic, metabolomic, and lipids, and two independent omics data for validation (proteomic and metabolomic). We used the 4-omics data from [6], collected from 128 adult SARS-CoV-2 virus-associated patients from Albany Medical Center in Albany, New York, USA. Blood samples of these patients were collected, and then transcripts, proteomics, metabolites, and lipids were measured from plasma. In addition, the authors identified and quantified the abundance of more than 17,000 different biomolecules (13,263 transcripts, 110 metabolites, 517 proteins, 646 lipids, and 2,786 unidentified small molecule signatures) in these 128 patient samples and recorded the omic data and clinical information. For severity prediction, we selected omic data and clinical information of 102 patients who presented positive tests for the SARS-CoV-2 virus.

In the four data cohorts, females accounted for 37.3% of the COVID-19 group. The mean age of patients in the COVID-19 group was 61.3 years for males and 63.1 years for females. The COVID-19 group has more ethnic diversity, with whites accounting for 46% of the total. These demographics in this

dataset are similar to the racial and ethnic health distribution reported by another study of COVID-19 [7]. The demographic distribution and characteristics of the dataset are similar to the distribution of social groups. So the dataset we use is somewhat representative of the proportion of healthy people, COVID-19 patients, and severe COVID-19 patients in society, with their demographic characteristics and health status.

2.1. Validation dataset

Independent datasets were used to validate the predictive performance of key features derived from the severity subgroup analysis. It can determine the method's robustness and assess generalisability to a wide range of omic data. Therefore, our selection criteria for the independent dataset were that it had to be the same omic type as the training dataset and be derived from a different sample of cases. Accordingly, we selected the proteomic and metabolomic data from the single-omic COVID-19 diagnosis problem. This dataset was used to verify the performance of the key features of proteomics and metabolomics obtained in training the four-omics model for predicting COVID-19 severity. We used the proteomics and metabolomics data with key features from the training set to train the model and verified them on the two validation data. We performed preliminary data processing. We retain the key features selected on the training set for the validation dataset. For the treatment of nulls in the dataset, we removed features with NA values greater than 20% and performed zero-value padding.

References

[1] J. Balnis, A. Madrid, K. J. Hogan, L. A. Drake, H. C. Chieng, A. Tiwari, C. E. Vincent, A. Chopra, P. A. Vincent, M. D. Robek, et al., Blood dna methylation and covid-19 outcomes, Clinical epigenetics 13 (1) (2021) 1–16.

[2] W. Liang, H. Liang, L. Ou, B. Chen, A. Chen, C. Li, Y. Li, W. Guan, L. Sang, J. Lu, et al., Development and validation of a clinical risk score to

predict the occurrence of critical illness in hospitalized patients with covid-19, JAMA internal medicine 180 (8) (2020) 1081–1089.

[3] Y. Lévy, A. Wiedemann, B. P. Hejblum, M. Durand, C. Lefebvre, M. Surénaud, C. Lacabaratz, M. Perreau, E. Foucat, M. Déchenaud, et al., Cd177, a specific marker of neutrophil activation, is associated with coronavirus disease 2019 severity and death, Iscience 24 (7) (2021) 102711.

[4] Y. Zheng, Y. Zhang, H. Chi, S. Chen, M. Peng, L. Luo, L. Chen, J. Li, B. Shen, D. Wang, The hemocyte counts as a potential biomarker for predicting disease progression in covid-19: a retrospective study, Clinical Chemistry and Laboratory Medicine (CCLM) 58 (7) (2020) 1106–1115.

[5] B. Shen, X. Yi, Y. Sun, X. Bi, J. Du, C. Zhang, S. Quan, F. Zhang, R. Sun, L. Qian, et al., Proteomic and metabolomic characterization of covid-19 patient sera, Cell 182 (1) (2020) 59–72.

[6] K. A. Overmyer, E. Shishkova, I. J. Miller, J. Balnis, M. N. Bernstein, T. M. Peters-Clarke, J. G. Meyer, Q. Quan, L. K. Muehlbauer, E. A. Trujillo, et al., Large-scale multi-omic analysis of covid-19 severity, Cell systems 12 (1) (2021) 23–40.

[7] M. W. Hooper, A. M. Nápoles, E. J. Pérez-Stable, Covid-19 and racial/ethnic disparities, Jama 323 (24) (2020) 2466–2467.
